# Pilocarpine-Induced Status Epilepticus Is Associated with P-Glycoprotein Induction in Cardiomyocytes, Electrocardiographic Changes, and Sudden Death

**DOI:** 10.3390/ph11010021

**Published:** 2018-02-16

**Authors:** Jerónimo Auzmendi, Bruno Buchholz, Jimena Salguero, Carlos Cañellas, Jazmín Kelly, Paula Men, Marcela Zubillaga, Alicia Rossi, Amalia Merelli, Ricardo J. Gelpi, Alberto J. Ramos, Alberto Lazarowski

**Affiliations:** 1 Laboratorio de Neuropatología Molecular, Instituto de Biología Celular y Neurociencia “Profesor E. De Robertis” IBCN UBA-CONICET, Buenos Aires CP1121, Argentina; jeronimo.auzmendi@gmail.com (J.A.); ivanhoe_rowena@hotmail.com (A.R.); jramos@fmed.uba.ar (A.J.R.); 2 Departamento de Patología, Instituto de Fisiopatología Cardiovascular (INFICA), Universidad de Buenos Aires, Facultad de Medicina, Buenos Aires C1121ABG, Argentina; brunobuchholz@yahoo.com.ar (B.B.); jazminkelly@hotmail.com (J.K.); rjgelpi@gmail.com (R.J.G.); 3 Departamento de Fisicomatemática, Laboratorio de Radioisótopos, Cátedra de Física, Universidad de Buenos Aires, Facultad de Farmacia y Bioquímica, Junín 956, Buenos Aires C1113AAD, Argentina; jsalguei@yahoo.com (J.S.); marcelazubillaga@yahoo.com (M.Z.); 4 Laboratorio Tecnonuclear SA, Arias 4176, Buenos Aires C1430CRP, Argentina; canellas@tecnonuclear.com; 5 Departamento de Bioquímica Clínica, Instituto de Investigaciones en Fisiopatología y Bioquímica Clínica (INFIBIOC), Universidad de Buenos Aires, Facultad de Farmacia y Bioquímica, Junín 956, Buenos Aires C1113AAD, Argentina; men.paula@hotmail.com (P.M.); amerelli2002@yahoo.com.ar (A.M.)

**Keywords:** Status Epilepticus, SUDEP, HIF-1α, hypoxia, P-glycoprotein, cardiomyocytes, QT prolongation

## Abstract

Sudden unexpected death in epilepsy (SUDEP) is the major cause of death in those patients suffering from refractory epilepsy (RE), with a 24-fold higher risk relative to the normal population. SUDEP risk increases with seizure frequency and/or seizure-duration as in RE and Status Epilepticus (SE). P-glycoprotein (P-gp), the product of the multidrug resistant *ABCB1-MDR-1* gene, is a detoxifying pump that extrudes drugs out of the cells and can confer pharmacoresistance to the expressing cells. Neurons and cardiomyocytes normally do not express P-gp, however, it is overexpressed in the brain of patients or in experimental models of RE and SE. P-gp was also detected after brain or cardiac hypoxia. We have previously demonstrated that repetitive pentylenetetrazole (PTZ)-induced seizures increase P-gp expression in the brain, which is associated with membrane depolarization in the hippocampus, and in the heart, which is associated with fatal SE. SE can produce hypoxic-ischemic altered cardiac rhythm (HIACR) and severe arrhythmias, and both are related with SUDEP. Here, we investigate whether SE induces the expression of hypoxia-inducible transcription factor (HIF)-1α and P-gp in cardiomyocytes, which is associated with altered heart rhythm, and if these changes are related with the spontaneous death rate. SE was induced in Wistar rats once a week for 3 weeks, by lithium-pilocarpine-paradigm. Electrocardiograms, HIF-1α, and P-gp expression in cardiomyocytes, were evaluated in basal conditions and 72 h after SE. All spontaneous deaths occurred 48 h after each SE was registered. We observed that repeated SE induced HIF-1α and P-gp expression in cardiomyocytes, electrocardiographic (ECG) changes, and a high rate of spontaneous death. Our results suggest that the highly accumulated burden of convulsive stress results in a hypoxic heart insult, where P-gp expression may play a depolarizing role in cardiomyocyte membranes and in the development of the ECG changes, such as QT interval prolongation, that could be related with SUDEP. We postulate that this mechanism could explain, in part, the higher SUDEP risk in patients with RE or SE.

## 1. Introduction

Patients with Refractory epilepsy (RE) or Status Epilepticus (SE) can develop a wide spectrum of cardiovascular events after severe convulsive seizures. These cardiac alterations include several electrocardiography abnormalities with malignant arrhythmias, asystole, infarct, or even sudden unexpected death in epilepsy (SUDEP), which is one of the most frequent causes of death among these RE patients [1,2,3]. SUDEP has been defined as a type of death related with epilepsy in which post-mortem anatomical and histological examination cannot disclose a clear cause of death [4,5]. Several conditions were described as risk factors for SUDEP, among which we can mention the high frequency of generalized seizures without control, mainly in patients aged between 20 and 40 years, or non-compliance with the specific medications that leads to a lack of seizure control. Interestingly, these situations can be observed in patients with RE receiving polytherapy where one of the pharmacoresistant mechanisms is likely related to high P-glycoprotein (P-gp) brain expression [6,7,8]. In this regard, higher risk factors of SUDEP, such as the high frequency of generalized tonic-clonic seizures (GTCS) and nocturnal seizures or a long history (more than 15 years) of seizures, have been observed mainly in patients with a pharmacoresistant phenotype. So, when the seizure frequency increases, the risk of SUDEP also increases [9], and this occurs particularly in patients after SE or severe RE under polytherapy. 

Previously, we described that daily induced-seizures with pentylenetetrazole (PTZ) increase P-gp expression in the hippocampus, and it is related to increasing membrane depolarization. This membrane depolarization cannot be reverted by phenytoin (PHT), but it was reverted when nimodipine, a calcium channel and P-gp blocker, was added [10]. Accumulated SE episodes in these animals lead to increased P-gp expression in the brain and heart, and ended with a fatal SE episode in all rats [11]. Taking into account that a progressive depolarization of the plasma membranes may affect myocardial function, we hypothesized that a fatal acute heart rhythm alteration could be the consequence of a severe hypoxic stress produced by the SE, where overexpressed cardiac P-gp plays the mentioned depolarizing role. Furthermore, experimental studies with animal models have described autonomic dysregulation with altered cardiac repolarization during and between seizures that could be the cause of SUDEP. These studies suggest that a harmful brain-heart connection could start from severe convulsive stress, inducing an acute heart deficiency and ending with a fatal cardiac arrhythmia [12,13,14,15]. 

Clinical and experimental studies have widely demonstrated that brain hypoxia-ischemia can induce seizures or epilepsy [16,17,18,19,20]. However, whether repetitive episodes of severe seizures as well as SE could induce chronic brain or systemic hypoxia-ischemia have been poorly investigated [21,22]. In this regard, one very complete experimental study confirmed the brain-heart connection in a model of SE induced by kainate. In this study, animals developed a cardiomyopathy, characterized by heart dysfunction with immediate elevation in plasma of noradrenaline, decreased ejection fraction, QT prolongation, and histological changes. Furthermore, this dilated cardiomyopathy, including cardiomyocyte vacuolization, apoptotic cells, cardiac fibrosis, and macrophages infiltration, was evident within 48 h of seizure induction and remained present for up to 28 days after of SE induced by kainate [23]. However, in this study, the role of hypoxia-inducible factor 1α (HIF-1α) and P-gp were not investigated. 

HIF-1α is the master transcriptional regulator of cellular and developmental response to hypoxia in all tissues. Consequently, oxygen deprivation in the brain and heart induces stabilization and nuclear translocation of HIF-1α and binding to the HRE-responsive genes that become upregulated. Two examples of the HRE-responsive genes are the erythropoietin receptor and P-gp (ABCB1/MDR-1) gene [24,25,26,27,28]. Based on the previously mentioned brain-heart connection, we speculate that severe and repetitive convulsive episodes or SE could generate a hypoxic-ischemic altered cardiac rhythm (HIACR), which should induce a concomitant heart expression of HIF-1α and P-gp.

One of the best characterized experimental models of SE in rats is produced by intraperitoneal (i.p.) administration of Li-Pilocarpine. This treatment induces SE followed by a silent period (~30 days post-SE), after which the spontaneous seizures are established. Furthermore, in our laboratory, it has been recently demonstrated that SE induced by Li-Pilocarpine also induces a parallel HIF-1α and P-gp expression in the brain [29]. This SE model, in turn, resembles the alterations that occur in patients with temporal lobe epilepsies [30], characterized by their pharmacoresistant phenotype and high hippocampal expression of P-gp [31].

Based on these observations, we aimed to verify whether the model of Li-Pilocarpine-induced SE produces a cardiac hypoxic condition with high expression of HIF-1α and P-gp in cardiomyocytes associated with electrocardiographic (ECG) alterations, and whether these findings are related to an increased spontaneous death rate. 

## 2. Results

### 2.1. SE Strength Induces Heart Rate Variation

**Figure 1:**
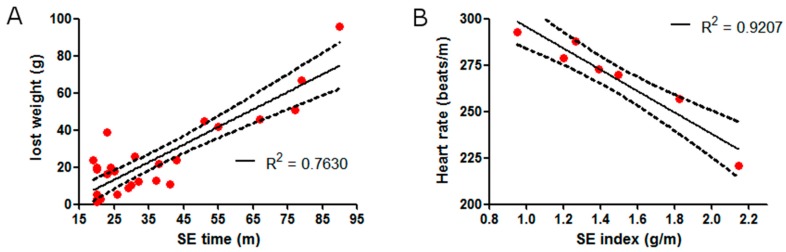
Strength of single Status Epilepticus (SE). (**A**) SE strength index. A linear correlation between SE time and lost weight 3 days after SE was observed. Note that the survival of the rats decreased with longer SE time. (**B**) Inverse correlation between SE strength index and heart rate variation is observed. The correlation’s coefficient (R^2^) and the confidence interval (dashed line) are showed in both graphics.

SE was induced by the Li-pilocarpine paradigm and was adjusted at 15, 30, 45, 60, 75, or 90 min of duration with diazepam, and weight loss was observed after each SE in all rats. To know the strength of the SE, we created the “SE-strength index”, which related the SE duration (in minutes) with post-SE weight loss, and a positive correlation between the post-SE weight loss and the SE duration was found (Figure 1A). The SE-strength index was calculated with a linear regression of R^2^ = 0.763. 

After a recovery period, heart rate was evaluated with a standard ECG. A high inverse linear correlation between heart rate and the SE-strength index (R^2^ = 0.9207) was observed, suggesting that the heart rate reduction was increased according with SE severity (Figure 1B). 

### 2.2. ECG Changes after Single SE

**Figure 2:**
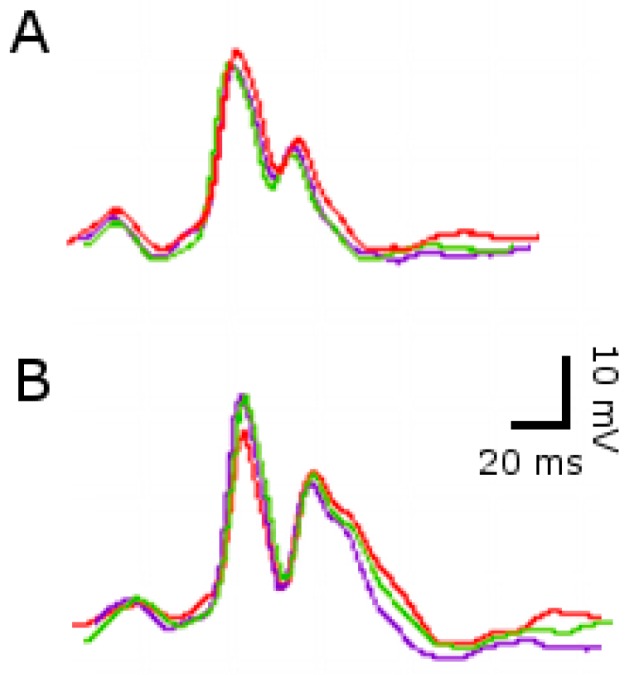
Representative traces of ECG after single SE. Three traces for each experimental group are shown. (**A**) ECG of the control group (No SE). (**B**) ECG of the convulsive group (SE). Note the elongated QT time in (**B**).

First, we measured the heart electrical function after the induction of only one SE episode. In the SE-group a decreased heart rate was observed. Additionally, QT time of the SE-group was also elongated by 15–20%, while PR interval and QRS amplitude post-SE remained unchanged. Because the QT time depends on the heart rate, we also analyzed the QT time with the normalized correction of Bazett´s formula (QTc), which indicated that after SE, QTc was increased independently of the heart rate (Table 1, Figure 2).

**Figure 3:**
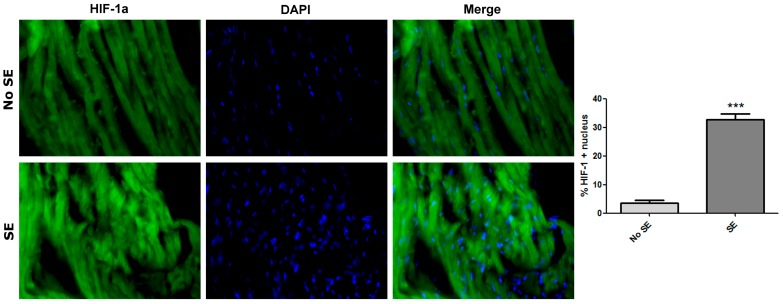
Cardiac hypoxia-inducible transcription factor (HIF)-1α expression. Micrographs (20×) of the myocardium expressing HIF-1α after SE and their quantification. Bars are the mean ± SEM. Differences were analyzed by Student *t*-test with a significance level of *p* < 0.001 (***).

**Figure 4:**
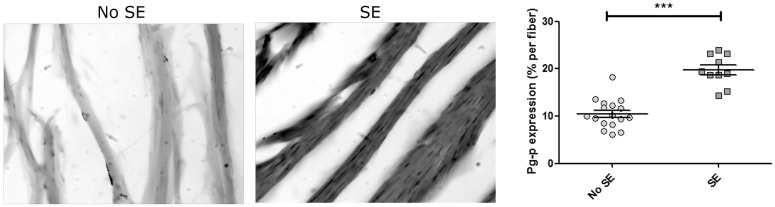
P-gp expression in myocardiocytes. Representative picture (20×) of the myocardiac fiber with or without SE and their respective quantitation. Values are given as mean ± SEM. Differences were analyzed by Student *t*-test with significance level of *p* < 0.001 (***).

Because seizures are assumed to be hypoxic-ischemic events, we evaluated the stabilization of HIF-1α and the P-glycoprotein expression in cardiomyocytes by immunohistochemistry in rats sacrificed 72 h after SE. HIF-1α was found stably expressed in the cytoplasm and translocated to the nucleus of cardiomyocytes at 3 days post-SE (Figure 3), and P-gp was expressed as patches in the membrane of the cardiomyocytes (Figure 4). All these data demonstrate that the repetitive convulsive stress promoted a persistent heart hypoxic stimulus, resulting in high expression of P-gp in cardiomyocytes associated with an altered electric cardiac function.

### 2.3. ECG Changes after Multiple SE

**Figure 5:**
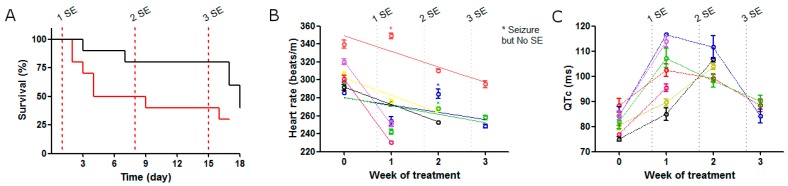
Analysis of multiple SE. (**A**) Survival after three weeks of treatment with SE of 15 min (black line, *n* = 10) or SE of 20 min (red line, *n* = 10). (**B**) Heart rate after each SE. Values are shown as mean ± standard deviation while lines are linear regression. Note that the heart rate was recovered when rats had seizures but had no SE (red, green and blue asterisks). (**C**) QTc-after each SE. Values are shown as mean ± standard deviation. For each plot, dashed lines represent the day of the SE.

We hypothesized that SEs result in hypoxic-ischemic events which induce stress in cardiomiocytes that, as a consequence, can increase the risk of SUDEP. To evaluate this, two different durations of SE induced by pilocarpine (15 or 20 min) were used, and these were repeated every seven days for three weeks, and the rate of spontaneous death in each group was recorded. The group that received 15 min of SE showed high survival (80%) in the first episode, but survival decreased to 50% in the third convulsive event. Conversely, the group exposed to 20 min of SE, showed a higher drop (50%) in survival after the first SE and a lower death rate (30%) in the last episode. However, at the end of the experiment, strikingly, both groups appeared to share the same rate of total spontaneous deaths (Figure 5A).

Next, in the group that underwent 20 min of SE, we evaluated the electrical heart function with ECG after each SE. The heart rate decreased after each SE (Figure 5B). Interestingly, three rats that had a seizure but not an SE recovered their normal heart rate by 72 h after treatment (Figure 5B asterisk). Additionally, the QT time was elongated after the first SE (Figure 5C). Strikingly, the same three rats, not only recovered QT time to reach the basal level at the third SE, but also survived, while all rats that increased the QT time died. All these data suggest that the higher risk of sudden death could depend on SE duration correlated with a lower heart rate and a greater QT elongation.

## 3. Discussion

Our study documents that weekly induction of SE for several weeks constitutes a model of convulsive stress with persistent ECG changes, characterized by prolongation of the QT interval and a high spontaneous death rate. The mechanism by which severe and repetitive convulsive stress could result in potentially fatal cardiac dysfunction is not fully understood. We speculate that prolonged generalized tonic-clonic seizures reaching the status epilepticus level should be assumed to be a severe hypoxic systemic stress.

A complex bidirectional interaction between the brain and heart exists, and in this context, prolonged or repetitive seizures could induce the activation of several hypoxia responsive genes in the heart. The most important modulator of these genes is the hypoxia-inducible factor-1α (HIF-1α), a transcription factor that is rapidly induced under hypoxia, and almost instantaneously degraded by proteasome when oxygen supply is normalized [28]. 

Because of this particular ability, the moderated number of cardiomyocytes expressing HIF-1α found at 72 h after the SE episode could be the consequence of a residual expression of HIF-1α activated during SE, but stabilized according to the remaining heart rate reduction. Interestingly, it is important to remark that HIF-1α evaluation was developed in rats during a period away from the acute convulsive episode. At this time, persistence of positive labelling for HIF-1α suggested that persistent bradycardia could be the cause of a chronic hypoxic injury affecting all organs and tissues (including brain and heart). So, the observed nuclear location of HIF-1α in cardiomyocytes is clear evidence that the heart was affected by hypoxia-ischemia during the SE episode and remained injured by the persistent heart rate reduction. In this context, a new event of excitotoxicity, perhaps not clinically evident as nocturnal seizures, could trigger a sudden death. In this regard, all rats were also found dead during a period away from the acute convulsive episode. 

All in all, repetitive convulsive stress such as SE, could be a systemic acute hypoxic insult strong enough to induce a hypoxic-ischemic altered cardiac rhythm (HIACR) [3]. Because the *ABCB1/MDR1* gene encoding the multidrug transporter P-glycoprotein (P-gp) is an HRE (HIF-1α) responsive gene [32], persistent HIF-1α expression in cardiomyocytes from convulsive rats (Figure 3) could explain why P-gp was robustly expressed in these cells (Figure 4). 

In a wide spectrum of experimental models of acute and chronic hypoxia in vivo, brain and heart expression of P-gp was previously demonstrated by us. In some of these studies, P-gp was simultaneously expressed with HIF-1α and another HIF-1α inducible gene as erythropoietin receptors (EPO-R) [25,33,34,35], where pharmacological nasal administration of human recombinant EPO protected the ischemic brain area [24]. Two other experimental studies of chronic and acute heart hypoxia-ischemia, developed in pigs and sheep, respectively, have demonstrated a significant loss of 99mTc-2-Methoxyisobutylisonitrile (^99m^Tc-SESTAMIBI) heart retention in the affected ischemic heart regions with a concomitant high expression of P-gp and the associated heart stunning [36,37]. These results could be the consequence of the active expression of P-gp, which not only plays a role in the radiotracer extrusion, but could also be directly involved in the intrinsic mechanism of cardiomyocytes membrane depolarization, as previously described in other cells [38,39,40,41]. Furthermore, in patients with dilated cardiomyopathy, an increased cardiac washout of ^99m^Tc-SESTAMIBI has been described, suggesting that this altered single-photon emission computed tomography (SPECT) study may predict mitochondrial dysfunction and impairment of myocardial contractile and relaxation functions during stress [42]. 

All this evidence reinforces our previous report showing that after repetitive seizures, the induced P-gp brain expression can contribute to cell membrane depolarization of the hippocampus and neocortex. In rats with repetitive seizures produced by daily doses of pentylenetetrazole (PTZ), a progressive phenytoin (PHT) resistant epileptic phenotype was observed, where an increased brain expression of P-gp was documented, and membranes were progressively depolarized. The recovery of a PHT-sensitive phenotype, and normalized membrane potential, were restored when PHT was administered together with nimodipine, a calcium channel blocker that also inhibits P-gp activity [10]. In this regard, we should point out that pioneer studies have demonstrated that P-gp can modify the resting membrane potential, producing depolarization with values from −70 to −10 mV [43,44]. In this regard, in a more recent preliminary experiment in rats developed under similar conditions as presented here, we can observe an important reduction of ^99m^Tc-SESTAMIBI heart retention 72 h after pilocarpine-induced SE [45]. This experiment was not developed to follow-up on heart alterations by dilated cardiomyopathy previously described after kainate-induced SE [23], however, our results could predict this alteration as mentioned above [42]. This preliminary experiment should be confirmed by more SPECT-stdies; its results are a clear demonstration that P-gp expressed in cardiomyocytes is active. In a purely speculative scenario, we believe that a range or zone of values showing “low ^99m^Tc-SESTAMIBI heart retention” could reveal the hypoxic-induced membrane depolarization in cardiomyocytes related with heart dysfunction after SE. This last condition could explain a persistent HIACR secondary to the convulsive stress with a later spontaneous sudden death as the SUDEP observed in patients underlying SE or severe refractory epilepsies. 

One intriguing characteristic of the current study is the high rate of spontaneous death, observed more than 48 h after each SE episode, which increased when the convulsive burden by repetitive induced SE accumulated. This particular feature resembles our previously mentioned studies where repetitive seizures induced in two independent experiments using two different seizure-inducers ended with fatal status epilepticus, and in both cases a progressive increase of P-gp expression in brain and heart was documented [8,11]. 

It is wildly accepted that acute myocardial ischemia results in greater membrane depolarization (less negative resting potential), which alters the cellular electrophysiology and the conduction of action potentials within the heart; these changes are reflected in the ECG pattern [46,47]. Within these alterations, the QT interval that represents the overall time required for initiation and completion of ventricular depolarization and repolarization can be longer, indicating membrane depolarization that is clinically reflected as bradycardia. Interestingly, in our study, the heart rate reduction got worse with successive SE and was associated with a higher spontaneous death ratio. Additionally, the heart rate reduction progressed with successive SE and was associated with a higher spontaneous death ratio. We postulate that the prolongation of the QT interval leads to spontaneous death, related to the continuous depolarizing function of P-gp in cardiomyocytes aggravated after each SE (Figure 4).

To date, the role of P-gp on membrane potential of hypoxic-ischemic cardiomyocytes has not been described. Is P-gp contributing to membrane depolarizations of cardiomyocytes after seizure-related severe hypoxia, as we previously described in hippocampus and neocortex? In the clinical setting, the most common cardiac response observed in both adults and children after complex partial crisis, as well as generalized tonic–clonic seizures, is increased heart rate in nearly 90% of the cases [48], while bradycardia or asystole related to seizures are very rare [47]. However, some studies have demonstrated that seizure-related bradycardia could be an important feature in patients with refractory epilepsy, and this heart failure could be associated with a higher risk of SUDEP [49,50,51,52]. 

It was reported that 40% of patients with refractory focal epilepsy had seizure-related ST-segment depression, suggesting that cardiac ischemia might occur during seizures [21]. However, in a complementary study from the same group of researchers, the patients with complex partial or generalized tonic–clonic seizures did not have elevated cardiac troponin levels after these crises [53]. Both reports suggest that heart ischemic episodes observed after seizures could be related to dysfunctional electrical properties more than with damaged cardiac tissue. Furthermore, a meta-analysis evaluating the heart rate variability (HRV) supports the hypothesis that autonomic cardiac dysfunction might play a role in SUDEP etiology, mainly in cases with low heart rate variability and bradycardia, or reduced vagal activity. This study also highlights that HRV can be a predictor of cardiovascular morbidity and mortality in patients with previous heart conditions [54]. Because the HRV index can be easily determined, it was recently suggested to be a useful predictive tool for the indication of vagal nerve stimulation as an alternative treatment of refractory epilepsy, in addition, it showed protective effects on the heart [55,56]. According to these descriptions, in our experiment, the observed heart rate reduction was not only sustained 24–48 h after each SE episode and associated with an increase of QT interval, but also was progressively more severe when the seizure load was increased, and it was associated with an increase in the death rate.

Our results, appear to be in accordance with a very recent communication that demonstrates a simultaneous P-gp overexpression in brain and peripheral tissues after ischemic stroke in rats [57]. Here, we are reporting for the first time that SE induced a sustained HIF-1α nuclear translocation driving a high P-gp expression in cardiomyocytes, which can contribute to their membrane depolarization, developing the ECG alterations with persistent a severe heart rate and prolonged QT interval. These conditions create a favorable scenario where new convulsive stress has a high possibility of inducing a sudden fatal heart failure, produced outside of the acute convulsive episode as observed in our study, and we assumed as a SUDEP experimental model. 

## 4. Materials and Methods

### 4.1. Ethics Statement

All procedures involving animals and their care were conducted in accordance with our institutional guidelines, which comply with the National Institutes of Health (NIH) guidelines for the Care and Use of Laboratory Animals and the principles presented in the Guidelines for the Use of Animals in Neuroscience Research by the Society for Neuroscience, and were approved by the institutional committee for the care and use of experimental animals (CICUAL) of the School of Pharmacy and Biochemistry, University of Buenos Aires. All efforts were made to minimize animal suffering and to reduce the number of animal used. 

### 4.2. Status Epilepticus Protocol

Status Epilepticus was induced in Wistar rats of 300 g using the Lithium-Pilocarpine paradigm [29], plus Diazepam (DZP) to stop the crisis. Briefly, SE was induced by i.p. administration of lithium (127 mg/kg), and 20 h later, pilocarpine (30 mg/kg). Severity of seizures was evaluated according to the Racine scale [58], and the rats were considered to have suffered SE when they remained for more than 5 min with myoclonic and/or tonic-clonic generalized seizures (stages 4 and 5 of said scale); DZP (20 mg/kg, i.p.) was given at different times after SE according to the experiment. All animals were monitored for the recovery period (24 h after each SE) in which they received 0.5 mL of physiological solution (i.p) every 6 h to maintain hydration. The control non-convulsive group was injected with a Lithium-saline solution. Once SE per week was induced, a single SE or multiple SE experimental schemes were used.

### 4.3. Experimental Scheme

Two different schemes were used. First, a single SE was induced and 24 h after the recovery period their heart function was evaluated and compared to that of a control group. Second, one SE per week was induced over three weeks. The basal ECG was studied in all animals previously to the induction of the first SE. After 24 h of each recovery period we evaluated the heart function, and then we analyzed the evolution compared to the basal ECG. We recorded sudden death after each SE, but excluded dead rats during SE or recovery period. In both experimental schemes, rats were allowed to freely convulse in room air. 

### 4.4. Electrocardiographic and Heart Rate Evaluation

Both control and convulsive rats were anesthetized with a mixture of Ketamine (35 mg/kg) and xylazine (5 mg/kg) administered intraperitoneally, as mentioned, to achieve a mild anesthesia. A standard ECG with needle-shaped electrodes placed subcutaneously in the four limbs was performed. After 5–10 min of stabilization period, a baseline ECG recording was taken. Simultaneously, the heart rate was evaluated from the length of the cardiac cycle (RR interval duration), the duration of the PR interval from the start of the P wave to the start of the QRS complex, and the duration of the QT interval from the start of the QRS to the end of the T wave. All variables were measured in five cycles in the DII derivative, and the arithmetic mean of each one was calculated. The measured QT interval values were corrected using the (QTc = QT/(RR) 1/2) and the normalized Bazzet equation (QTcn = QT/(RR/f) 1/2), where f is the factor of normalization that arises from values of the basal RR intervals [59]. 

### 4.5. Immunohistochemistry

After deep anesthesia with ketamine/xylazine (90/10 mg/kg), animals were sacrificed by intracardiac perfusion with 4% paraformaldehyde in phosphate buffer as previously described [29]. Frozen hearts were cut into 14-micron sections and mounted on slides. Subsequently, the sections were dehydrated to perform the inhibition of endogenous peroxidase with H_2_O_2_ (0.5% *v*/*v* in methanol) for 30 min at room temperature; then rehydrated and permeabilized with 1% Triton X-100 in buffer phosphate saline (PBS) for 30 min. Nonspecific sites were blocked with 3% equine normal serum in PBS for 30 min, and incubation with anti-P-gp (dilution: 1/500; EPR10364-57 Abcam Inc., Cambridge, MA, USA) was performed for 48 h at 4 °C. Subsequently, incubation with a biotinylated secondary antibody (dilution 1/800; B7389 Sigma, St. Louis, MO, USA) was performed for 3 h at room temperature. Immunoreactivity was revealed with diaminobenzidine/nickel until coloration was observed and mounted with DPX (Distrene-Plasticizer- Xylene) mounting medium. Slides were also treated with monoclonal antibody anti-HIF-1α (dilution: 1/500 NB-100-131 NovusBio, Littleton, CO, USA) and revealed by immunofluorescence using a labelled Alexa488 donkey anti-mouse (Jackson ImmunoResearch Laboratories, Inc., West Grove, PA, USA). 

## 5. Conclusions

We postulate that SUDEP can be induced by SE or highly frequent severe generalized seizures themselves, and it must be assumed to be a severe hypoxic event. SUDEP is a process related to the cumulative burden of convulsive stress that progressively will install the pharmacoresistant phenotype. Another consequence of an induced seizure is the simultaneous high expression of P-gp in the brain and heart. Under these considerations, our results suggest that increased P-gp expression in the heart contributes to the membrane depolarization of cardiomyocytes resulting in an acute and fatal alteration of heart rhythm. These experimental results could help to explain, in part, the particularly high frequency of SUDEP observed in RE patients, in whom P-gp can be over expressed. In these patients with repetitive generalized-seizure stress, a high cumulative burden of heart hypoxia-ischemic condition can induce heart P-gp expression, leading to an epileptic-related fatal HIACR, as a mechanism of SUDEP.

## Figures and Tables

**Table 1:** Electrocardiograph (ECG) after one SE. Table shows the most common parameters of an ECG to control group (No SE) and convulsive group (SE). QTc parameter represents QT time corrected by the Bazzet normalized equation. The values are listed as mean ± standard error and differences were analyzed by Student *t*-test with *p* < 0.05 (*).

ECG	No SE (*n* = 3)			SE (*n* = 7)		
Heart rate (beats/m)	321	±	8.64	271	±	9.03 *
RR interval (ms)	187	±	5.05	223	±	8.32 *
PR interval (ms)	50.5	±	0.29	49	±	1.22
QRS amplitude (mV)	27.3	±	2.19	27.1	±	1.03
QT (ms)	72	±	0.50	85.3	±	3.74 *
QTc (ms)	71.9	±	1.60	85.5	±	3.68 *
